# Routine processing procedures for isolating filamentous fungi from respiratory sputum samples may underestimate fungal prevalence

**DOI:** 10.3109/13693786.2011.615762

**Published:** 2011-09-30

**Authors:** Catherine H Pashley, Abbie Fairs, Joseph P Morley, Shreeya Tailor, Joshua Agbetile, Mona Bafadhel, Christopher E Brightling, Andrew J Wardlaw

**Affiliations:** Institute for Lung Health, Department of Infection, Immunity and Inflammation, University of Leicester, Leicester, UK

**Keywords:** *Aspergillus fumigatus*, yeast, culture methods, respiratory samples, fungal growth media

## Abstract

Colonization of the airways by filamentous fungi can occur in asthma, chronic obstructive pulmonary disease (COPD) and cystic fibrosis. A recent study found IgE sensitization to *Aspergillus fumigatus* to be associated with reduced lung function. Significantly higher rates of *A. fumigatus* were detected in sputum from asthmatics sensitized to this fungus compared to non-sensitized asthmatics. The rate of positive cultures was far higher than equivalent historical samples analysed by the local clinical laboratory following protocols recommended by the UK Health Protection Agency (HPA). This study compares the HPA procedure with our sputum processing method, whereby sputum plugs are separated from saliva and aliquots of approximately 150 mg are inoculated directly onto potato dextrose agar. A total of 55 sputum samples from 41 patients with COPD were analyzed, comparing fungal recovery of five dilutions of sputa on two media. Isolation of A. *fumigatus* in culture was significantly higher using the research approach compared to the HPA standard method for mycological investigations (*P* < 0.001). There was also a significant difference in the recovery rate of *A. fumigatus (P* < 0.05) between media. This highlights the need for a standardized approach to fungal detection which is more sensitive than the method recommended by the HPA

## Introduction

Airway diseases such as asthma, chronic obstructive pulmonary disease (COPD) and cystic fibrosis (CF) are common, important causes of disease and ill health. Colonization of the airways by filamentous fungi can occur in all three disease groups, although the clinical relevance is unclear. Allergic bronchopulmonary aspergillosis (ABPA) is well recognized as a severe complication of airway colonization associated with a florid hypersensitivity reaction to *Aspergillus fumigatus* reported in up to 8% of asthmatics [1] and 13% of CF patients [2]. Fungal colonization may have a deleterious effect without fulfilling all criteria necessary for a diagnosis of ABPA. One of the strongest risk factors of filamentous fungi in CF is decreased lung function, even after exclusion of patients diagnosed with ABPA. However, it is unclear whether fungal colonization contributes to lower lung function or is a marker of more severe lung disease and aggressive therapy. Incidence of recovery of at least one fungal species from an individual is around 40%, with prevalence rates having significantly increased in the last decade [3]. In contrast to CF [3, 4], there has been no comprehensive studies looking at fungal colonization in asthma or COPD. Those that exist mostly focus on patients suspected of having ABPA and primarily report only *A. fumigatus*.

Sputum samples are frequently used to study airway inflammation in respiratory diseases and to perform microbiological investigations of respiratory infections. In comparison to bronchoalveolar lavage (BAL), sputum offers the advantage of being non-invasive to obtain, and is therefore more readily available and suitable for repeated measurements. The use of an induction protocol can result in samples being obtained from over three quarters of normal and asthmatic subjects who cannot produce sputum spontaneously [5].

While mycology laboratory accreditation programs are common, most countries including the USA, Canada and Australia, have no national standard guidelines for processing respiratory samples. In the UK most National Health Service (NHS) clinical microbiology laboratories follow the national standard method set out by the Health Protection Agency (HPA) in BSOP57 [6].

A recent study by our group found IgE sensitization to *A. fumigatus* to be associated with reduced lung function in asthma. In addition, significantly higher rates of *A. fumigatus* were detected in sputum from *A. fumigatus*-IgE-sensitized asthmatics (63%) compared to non-sensitized asthmatics (31%) and healthy subjects (7%) [7]. One striking finding was the high rate of recovery of fungi in culture compared to the routine NHS clinical laboratory (using the HPA method); where on historical samples < 10% of patients had a positive culture. There were a number of differences in technique that could explain the disparity in culture prevalence, including quantity of inoculating material and media used for culture. The research and clinical samples were not taken at the same time so the results could not be directly compared.

The aim of this study was to directly compare our methodology with that recommended by the HPA, in particular comparing the effect of dilution of sputum on incidence of *A. fumigatus* positive cultures, and to determine the influence different media had on culture rates of *A. fumigatus* and yeast.

## Materials and methods

### Patients

Sputum samples were obtained from patients with COPD as they represent a cohort which readily produces the large volumes of to adequately compare the techniques. Samples were obtained from patients recruited from the general respiratory clinic at Glenfield Hospital (Leicester, UK) who had a physician's diagnosis of COPD according to the global initiative for chronic obstructive lung disease (GOLD) criteria [8] and were able to produce > 2 ml of sputum. Subjects were recruited as part of a study investigating the use of biomarkers to target therapy during exacerbations of COPD [9]. The study was approved by the Leicestershire, Northamptonshire and Rutland Ethics Committee, and all subjects gave informed written consent.

### Sputum induction, processing and identification of fungal isolates

Some patients produced sputum spontaneously, for others sputum induction was performed as described previously [5, 10]. Expectorated samples were stored on ice, and processed within 2 h within a class II hood. Quality of sputum was evaluated in accordance with previous work [11]. The sputum sample was divided into two parts, ensuring an approximately equal quantity of plug and saliva was distributed between aliquots. One part was used to obtain a homogenized sample, the other to obtain sputum plugs.

The HPA BSOP57 guidelines are designed for the identification of both bacteria and fungi from a single sample. For routine microbiological investigations that are not specifically designed to detect fungi, the sputum sample is homogenized, diluted, inoculated onto Sab-ouraud dextrose agar (SDA) containing 50 μ/ml chloramphenicol (SC) and incubated at 37°C for two days. For specific mycological investigations the samples were undiluted homogenized sputum and incubation period was increased to five days. The whole expectorate of sputum plug and saliva is used. In contrast, our routine approach sputum plugs are carefully removed from saliva prior to their inoculation onto potato dextrose agar (PDA) containing 16 μ/ml chloramphenicol, 4 μg/ml gentami-cin and 5 μ/ml fluconazole (PGCF). In studies of airway inflammation, careful selection of sputum plugs contributes to less salivary squamous cell contamination [5], leading to fewer bacterial species recovered in culture, indicating less oropharyngeal contamination [10]. The use of undiluted sputum was used previously to investigate the prevalence of *A. fumigatus* in patients with cystic fibrosis [12].

The sputum for homogenization was mixed with an equal volume of 0.1% DL-dithiothreitol (DTT) and incubated at 37°C for 15 min. After incubation, 10 μl of homogenized sputum was diluted 1/500 in sterile water. Aliquots (10 μl and 100 μl) of both homogenized and diluted-homogenized sputum were inoculated in parallel onto both PGCF and SC plates. For the second part, plugs were separated from saliva, and approximately 150 mg (100–250 mg) inoculated onto PGCF and SC plates. As an additional control, 100 μl of 0.1% DTT was inoculated onto both media. All plates were sealed with nescofilm and then transferred to a separate laboratory where they were incubated for seven days at 37°C. Plates were inspected, without opening, three times (after 40–18 h, between days 4 and 6, and on day 7) and the number of visible colonies recorded at each time point. After seven days, filamentous colonies were examined and *A. fumigatus* identified based on macroscopic and microscopic features [13].

### Statistical analysis

Matched data was analyzed by McNemar test or Cochran's Q test. A multiple logistic regression was performed to explore the factors relating to fungal culture. Explanatory variables included media (SC or PGCF), organism (yeast or *A. fumigatus*), and dilution of sputum. McNemar test was calculated using GraphPad's QuickCalcs web page at http://www.graphpad.com, Cochran's Q test and multiple logistic regression were performed using SPSS version 18. All *P*-values were two-tailed. *P*-values < 0.05 were considered as statistically significant.

## Results

A total of 55 sputum samples from 41 patients were analyzed. Each sample was obtained on a separate visit and was independently analyzed. A total of 10 culture plates were inoculated with each sample. Yeasts were isolated from ≥ 1 plate from all but one of the samples (98%) that were inoculated on ≥ 1 plate, and 29% of samples (16 of 55) yielded *A. fumigatus*.

There was a clear effect of diluting sputum on yeast recovery with both media (PGCF, Q= 129.381, df = 4, *P* <0.000; SC, Q = 137.654, df=4, *P* <0.000), with fewer patients being found to be positive with more dilute sputum (PGCF sputum plug 52/55 versus 10 μl diluted-homoge-nized sputum 9/55, χ^2^_(1) McNemar_ = 41.02, *P* = < 0.0001 (comparable data on SC); Table 1). There were no differences between recovery rates of yeasts from homogenized sputum compared to sputum plug (PGCF sputum plug 52/55 versus 10 μl homogenized sputum 48/55, χ^2^_(1)_ McNemar = 2.25, *P* = 0.1336 (comparable data on SC); Table 1).

**Table 1:** Distribution of plates positive for yeast growth (YP) from 55 sputum samples inoculated at various dilutions onto potato dextrose agar (PGCF) or Sabouraud dextrose agar (SC).

	Diluted-homogenized		Homogenized		Plug
			
Sputum	10 μl	100 μl	10 μl	100 μl	150 mg^1^
% YP PGCF plates	16.4	50.9	87.3	96.4	94.5
% YP SC plates	10.9	34.5	83.6	96.4	94.5
No. of YP samples on both media	2	17	45	52	51
No. of YP samples on PGCF only	7	11	3	1	1
No. of YP samples on SC only	4	2	1	1	1

1 range 100 – 250 mg.

Detection of *A. fumigatus* was highly dependent on quantity of sputum inoculated onto the culture plate (PGCF, Q = 33.667, df = 4, *P* < 0.000; SC, Q = 13.867, df = 4, *P* < 0.008). Of the 16 samples that were *A. fumigatus*-positive on > 1 plate, growth of the fungus was not detected with diluted-homogenized sputum samples (Table 2). Not considering differences due to the media, 19% and 44% of *A. fumigatus-positive* samples were detected using 10 μl and 100 μl of homogenized sputum, respectively, and 94% when a neat sputum plug was inoculated. One sample was culture negative with a sputum plug, but culture positive with 100 μl homogenized sputum.

**Table 2:** Number of *Aspergillus fumigatus* colonies isolated from COPD patient sputum plated onto either potato dextrose agar (PGCF) or Sabouraud dextrose agar (SC) at various dilutions, and the time period during which colony growth was fi rst observed.

		Number of *A. fumigatus* colonies isolated									
		
		diluted-homogenized				Homogenized				Plug	
				
		10 μl		100μl		10μl		100μl		150 mg 1	
						
Sputum Sample	Time period	PGCF	SC	PGCF	SC	PGCF	SC	PGCF	SC	PGCF	SC
1	7 days							1			
2	7 days									1	
3	4 – 6 days									1	
4	4 – 6 days							1		1	
5	4 – 6 days										1
6	40 – 48 h					1					1
7	40 – 48 h									1	
8	40 – 48 h							1	1	1	
9	40 – 48 h									2	
10	40 – 48 h									2	
11	40 – 48 h									2	
12	40 – 48 h									3	
13	40 – 48 h							4	1	2	7
14	40 – 48 h							4		28	
15	40 – 48 h					3	1	13	11	18	31
16	40 – 48 h					5	20	26	34	>40	>50
% positive (*n* = 16)		0	0	0	0	19	13	44	25	81	31

1 range 100 – 250 mg.

The proportion of plates that were culture positive for either yeast or *A. fumigatus* was either the same on the two media or higher on PGCF at all dilutions. Based on multiple logistic regression analyses, media (B = − 0.528, SE = 0.204, *P* = 0.010) dilution (B = 1.403, SE = 0.108, *P* = 0.000) and organism (B = − 5.333, SE = 0.331, *P* = 0.000) all independently had a significant effect, after correction for each other, on whether positive cultures were obtained.

**Fig. 1:**
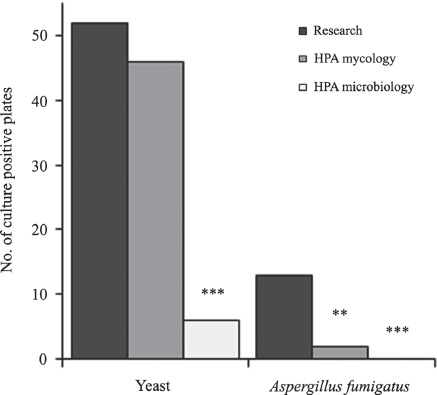
Number of culture positive plates detected using approximately 150 mg neat sputum plug plated directly onto PGCF plates (research) compared to the health protection agency (HPA) standard for mycological investigations (10 μl of homogenized sputum) or microbiological investigations (10 μl of diluted-homogenized sputum) plated onto SC plates, *n* = 55. **P <0.01, ***P < 0.001, HPA methods compared to research using McNemar test.

Sputum plugs inoculated directly onto PGCF resulted in the highest *A. fumigatus* detection rates (13 of 16). Directly comparing the two methodologies, the isolation of *A. fumigatus* was significantly higher using neat sputum plug on PGCF compared to the standard for mycological investigations (10 μl of homogenized sputum on SC; χ^2^_(1) McNemar_ = 9.09, *P* = 0.0026; Fig 1) or the standard for microbiological investigation not specifically targeting fungi (10 μl of diluted-homogenized sputum on SC; χ^2^_(1) McNemar_ = 11.08, *P* = 0.0009). Likewise, the recovery of yeasts was significantly higher with our research approach as compared to the standard for microbiological investigations (χ^2^_(1)__McNemar_ = 44.02, *P* = < 0.0001) but not compared to the standard for mycological investigations (χ^2^_(1) McNemar_ = 3.13, *P* = 0.0771).

In the majority of cases (11 of 16) filamentous fungal growth was observed after two days incubation at 37°C, with fungi detected in three samples after 4–6 days of incubation and growth was not detected with two until the 7th day.

## Discussion

Both the choice of media and quantity of sputum inoculated onto the tested media have an effect on the detection of fungi from respiratory samples. The primary aim of this study was to directly compare our research focused approach for the recovery of fungi from sputum specimens to the HPA recommended approach. If the samples obtained in this study had been referred for microbiological investigation without specifically requesting fungal culture, all would have been negative for *A. fumigatus.* This suggests that unless a clinician specifically suspects fungi as a cause of a respiratory problem, fungal colonization is likely to be missed. If a mycological investigation had been requested, only 12.5% of the ‘ever *A. fumigatus-positive’* samples would have been detected.

A higher starting quantity of sputum resulted in a clear increase in number of A. *fumigatus* colonies retrieved from three samples. The presence of fungi in the remaining samples was often detected on a single plate, and 50% resulted in the isolation of a single colony. It is unclear whether there is a clinically relevant difference between recovery of multiple colonies compared to a single colony. However, multiple colonies could be hypothesized as reflecting a longer term or more severe colonization event. Unfortunately counting colonies is not always feasible as *A. fumigatus* is fast growing, often appearing within a day or two of incubation and some colonies rapidly spread over the entire plate, particularly with PGCF media, preventing growth of additional colonies.

In our original asthma study [7], we assumed that the quantity of inoculating material was the prime factor accounting for differences between approaches, and stated that the other factor, i.e., choice of media, was less likely to have affected recovery rates. In this investigation we found isolation rates for yeasts and *A. fumigatus* were much higher on PGCF than SC, which was unexpected. This surprising result was particularly true for *A. fumigatus.* Both PDA and SDA are commonly used general-purpose mycological media. We selected PDA over SDA based on a comparison of conidial formation in pure cultures of seven allergenic species representing different fungal genera, i.e., *Alternaria, Aspergillus, Botrytis, Cladosporium, Epicoccum, Leptosphaeria,* and *Penicillium* (unpublished data). In all cases the fungi readily grew on both media, but a higher yield of conidia was obtained on PDA. We added antibiotics to the PDA at optimum concentrations for isolation of pathogenic fungi [14], and fluconazole to enhance recovery of *A. fumigatus* through suppression of *Candida* [15]. It is unclear as to whether *A. fumigatus* grows preferentially on PDA compared to SDA or the higher concentration of chloramphenicol in SC plates inhibits *A. fumigatus* development or the fluconazole enhances *A. fumigatus* recovery. Further testing would be required to address these issues, but this study highlights the fact that choice of media does make a difference and that SC, used routinely in many clinical mycological laboratories, may result in the underestimation of the prevalence of *A. fumigatus* colonization.

The HPA guidelines recommend two-day incubation of specimens for standard microbiology studies and five days for mycological analysis. In this study the fungus in the majority of *A. fumigatus* positive samples was observed within 2–5 days, although seven days of incubation was needed for fungal isolation with two samples. While *A. fumigatus* and yeasts are fast growing organisms some clinically relevant fungi grow more slowly. *A. fumigatus* is the most prevalent filamentous fungi isolated from respiratory samples in cases of CF [3,4,12,16], and asthma [J. Agbetile, unpublished results]. Once established *A. fumigatus* can spread to cover the entire plate preventing growth of other fungi. In the absence of *A. fumigatus,* samples may be incubated for longer periods. The HPA recommendation is that if *Paracoccidioides brasiliensis* infection is clinically indicated it is best to incubate samples for up to six weeks [6]. In these instance bijoux bottles are recommended instead of culture plates to prevent the media from drying out.

The clinical relevance of the isolation of fungi from sputum samples is still a matter of controversy, particularly with regard to more infrequently detected species. In CF, persistent fungal colonization of the airways is thought to exacerbate lung damage [16]. Studies looking at the treatment of CF-ABPA patients with antifungal agents have shown an increase in FEV_1_ as one of the outcomes [17], suggesting elimination of colonizing fungi leads to better lung function. In asthma, IgE-sensitization to fungi is common in individuals with severe disease, being reported in up to 66% of people with severe asthma in one study [18], and treatment with antifungal drugs has been shown to improve quality of life [19]. Furthermore, we have found lung function to be worse in *A. fumigatus*-IgE-sensitized asthmatics compared to non-sensitized asthmatics [7]. There is a growing body of evidence suggesting fungi may be having a deleterious effect in a far higher number of individuals than once suspected. To fully understand the link between fungi and ill health, and to determine the efficacy of treatments for eliminating fungi from the airways, more sensitive means of identifying fungal colonization are required.

This study aimed to directly compare our research method for culturing fungi from sputum samples to the HPA protocol followed by NHS clinical laboratories. We do not claim that our approach is the ideal way to isolate filamentous fungi from respiratory specimens. What this study has shown is that the approach most commonly used may be very insensitive. A recent multi-centre study looking at prevalence of fungi isolated from sputum of CF patients using different culture protocols has highlighted the need for a standardized approach to be adopted, and the pressing need for an optimal method for analysis of the fungal component of CF microbiology [16]. We echo that sentiment, but believe a standardized approach is required for all studies of fungal colonization of the respiratory tract, not just CF, and that a more sensitive approach is needed to truly understand the health impact of fungi.
